# Health Behavior Changes during the COVID-19 Pandemic: A Longitudinal Analysis among Children

**DOI:** 10.3390/ijerph19159220

**Published:** 2022-07-28

**Authors:** Traci A. Bekelman, Yanan Dong, Amy J. Elliott, Assiamira Ferrara, Kaylyn Friesen, Maren Galarce, Diane Gilbert-Diamond, Deborah H. Glueck, Monique M. Hedderson, Christine W. Hockett, Margaret R. Karagas, Emily A. Knapp, Maristella Lucchini, Julia C. McDonald, Katherine A. Sauder, Dana Dabelea

**Affiliations:** 1 Lifecourse Epidemiology of Adiposity and Diabetes (LEAD) Center, Anschutz Medical Campus, University of Colorado, Aurora, CO 80045, USA; kaylyn.friesen@cuanschutz.edu (K.F.); deborah.glueck@cuanschutz.edu (D.H.G.); katherine.sauder@ucdenver.edu (K.A.S.); dana.dabelea@ucdenver.edu (D.D.); 2 Department of Epidemiology, Johns Hopkins Bloomberg School of Public Health, Baltimore, MD 21205, USA; ydong29@jhmi.edu (Y.D.); eknapp2@jhu.edu (E.A.K.); 3 Avera Research Institute, Sioux Falls, SD 57108, USA; amy.elliott@avera.org (A.J.E.); christine.hockett@avera.org (C.W.H.); 4 Department of Pediatrics, School of Medicine, University of South Dakota, Sioux Falls, SD 57108, USA; 5 Kaiser Permanente Northern California Division of Research, Oakland, CA 94612, USA; assiamira.ferrara@kp.org (A.F.); maren.n.robinson@kp.org (M.G.); monique.m.hedderson@kp.org (M.M.H.); julia.c.mcdonald@kp.org (J.C.M.); 6 Department of Epidemiology, Medicine and Pediatrics, Geisel School of Medicine at Dartmouth, Hanover, NH 03755, USA; diane.gilbert-diamond@dartmouth.edu (D.G.-D.); margaret.r.karagas@dartmouth.edu (M.R.K.); 7 Department of Pediatrics, Anschutz Medical Campus, University of Colorado, Aurora, CO 80045, USA; 8 Department of Psychiatry, Columbia University Irving Medical Center, New York, NY 10032, USA; ml3551@cumc.columbia.edu

**Keywords:** longitudinal, COVID-19, disparities, diet, physical activity, screen time, sleep

## Abstract

This longitudinal study compared children’s health behaviors before the COVID-19 pandemic versus during the pandemic. This analysis examined the association between individual-level characteristics and health behavior change. Four prospective cohort studies in the Environmental influences on Child Health Outcomes (ECHO) Program contributed data. Children aged 4–12 years and their caregivers were recruited in California, Colorado, North Dakota, and New Hampshire. Dietary intake, physical activity, screen time, and sleep duration were assessed with questionnaires pre-pandemic and during the pandemic. The final sample included 347 children: 47% female and 62% non-Hispanic White. Compared with pre-pandemic, weekday screen time duration was higher during the pandemic (3.0 vs. 4.5 h, *p* < 0.001). Unadjusted increases in screen time duration differed by race and ethnicity: 1.3 h/day for non-Hispanic White children, 2.3 h/day for Hispanic children, and 5.3 h/day for non-Hispanic Black children. Overall, no changes occurred in sugar-sweetened beverage (SSB) intake (*p* = 0.26), discretionary food intake (*p* = 0.93), and physical activity (*p* = 0.15). Sleep duration increased by 30 min among children who did not meet sleep recommendations pre-pandemic. Child sex and maternal education level were not associated with health behavior change. The pandemic may have exacerbated disparities in some health behaviors. Families may need support to re-establish healthy routines.

## 1. Introduction

When the World Health Organization (WHO) declared COVID-19 a pandemic on 11 March 2020, public health precautions were implemented worldwide to prevent the spread of the SARS-CoV-2 virus [1]. Precautions, such as school closures and stay-at-home orders, were generally most restrictive in the initial lockdown period. The post-lockdown period was characterized by physical distancing precautions, a widespread paring down of events and gatherings and greater use of online platforms for many daily activities, although restrictions varied by location and over time [2] These societal changes are relevant for pediatric populations because children’s health behaviors are responsive to environmental conditions, and associated with their mental health, risk for cardiometabolic diseases, academic achievement, and quality of life [3,4,5,6,7,8] The extent of the public health precautions during the COVID-19 pandemic was nearly unprecedented and societal changes are ongoing; therefore, the impact on children’s health behaviors has not yet been fully investigated.

Among children in Europe, Asia, Australia, and the Middle East, dietary intake, physical activity, screen time, and sleep were altered by the pandemic. Screen time duration and consumption of discretionary foods (e.g., cakes and candy) increased [9,10,11,12], physical activity decreased [10,13,14], and bedtimes and wake-up times shifted to later [15,16]. Sugar-sweetened beverage (SSB) intake [9,10,17] and sleep duration [10,15,18] also changed, although the direction of change was not consistent across studies. Among United States (U.S.) children, one study mirrored these findings. However, the study [19] included repeated measures from only 74 children from one area; therefore, the findings may not reflect the broader pediatric population. Despite these studies, several gaps remain. Most studies measured health behaviors only during the earliest, most restrictive lockdown period and gave no information about the ongoing pandemic effects [20]. Several retrospective studies administered one survey during the pandemic to assess perceived change [21,22,23], but this approach could lead to misreporting if a respondent’s recall of pre-pandemic behaviors is inaccurate. More high-quality studies are needed [24,25].

The magnitude and direction of behavior changes may not be uniform across the population, although this has not been directly investigated among U.S. children. Cross-sectional studies conducted in the U.S. pre-pandemic [26,27,28] and during the first few months of the pandemic [29] show a relationship between health behaviors and individual child characteristics, including race/ethnicity, sex, age, and socioeconomic status. The pandemic has contributed to changes in household conditions that can influence health behaviors, such as parent employment, access to health-related services, and food insecurity [30,31,32]; although the groups most affected by the pandemic at the household level were not always those with historical disadvantage [33]. Additionally, novel household factors during the pandemic, including changes to parents’ work schedules to care for their children during remote schooling and pandemic-related financial concerns as a source of stress have also been linked to children’s health behaviors [29]. Thus, societal changes during the pandemic could have reduced or exacerbated disparities in health behaviors. The objective of this study among children aged 4 to 12 years in the U.S. was to compare dietary intake, physical activity, screen time duration, and sleep duration pre-pandemic (1 July 2019–1 March 2020) versus during the pandemic (1 December 2020–30 April 2021), with a focus on identifying children whose health behaviors were most impacted.

## 2. Methods

### 2.1. Study Design and Population

This longitudinal study followed a sub-set of caregiver-child dyads in the Environmental influences on Child Health Outcomes (ECHO) Program, a consortium of pediatric cohort studies collecting new data under a common protocol since 2019 [34,35]. Caregivers are referred to as parents in this manuscript; the term parent was used in the most inclusive sense. Single and cohort-specific institutional review boards monitored human subject activities and the ECHO Data Analysis Center. Participants provided informed consent.

This study was limited to cohorts that assessed health behaviors among children aged 4 to 12 years under the ECHO protocol in the 8-month period preceding the pandemic. The cohorts agreed to collect follow-up data from the same participants during the pandemic, although some cohorts only assessed 3 of 4 health behaviors pre-pandemic. The sample was limited to this age range because the environmental factors that influence health behaviors were expected to be different for toddlers and adolescents compared with children ages 4 to 12 [36,37,38]. Children’s dietary intake, physical activity, screen time, and sleep duration were assessed during the 8 months preceding the COVID-19 pandemic (1 July 2019–1 March 2020) as part of the ECHO protocol. Repeated measures of the same behaviors were collected during the pandemic (1 December 2020–30 April 2021) on the same dyads. Seasonal variation in health behaviors was not expected to be clinically relevant [39]. The sample included 347 children from cohorts with recruitment sites in 4 U.S. states. Families with the least amount of missing data pre-pandemic were prioritized for inclusion and recruited by phone, email, or text. Assessments were completed in-person at a research visit or remotely via child self-report or parent-proxy report.

### 2.2. Sociodemographic Characteristics

Sociodemographic variables were collected via self-report or medical record abstraction. Categorical variables included child sex assigned at birth, child race and ethnicity, annual household income, and highest level of maternal education. Child age was continuous. These sociodemographic characteristics were selected for inclusion because each characteristic was linked to child health behaviors pre-pandemic [26,27,28], and the Household Pulse Survey conducted in summer 2020 indicated that lower-income families and families who were Black or Hispanic, enduring greater economic hardship during the first few months on the pandemic [32].

### 2.3. Health Behaviors

Dietary intake was assessed with the Block Kids Food Frequency Questionnaire (FFQ) [40] or the Dietary Screener Questionnaire (DSQ) [41]. These questionnaires were developed and validated for assessing food intake among children aged 4 to 12 years (child self-report or parent-proxy report). The FFQ queried intake frequency and average portion size of 85 food and beverage items over the preceding week. The DSQ queried intake frequency of 25 food items or categories over the preceding month. Data were harmonized to produce 2 categorical variables reflecting intake frequency of SSBs and sugar-sweetened discretionary foods with 6 response categories: less than once/week, once/week, twice/week, 3–4 times/week, 5–6 times/week, and once/day or more. SSBs were defined as soft drinks; sweetened coffee, tea, or fruit drinks; and sports/energy drinks. Artificially sweetened beverages and 100% fruit juice were excluded. Flavored milks were excluded because they could not be distinguished from unflavored milks in the DSQ. Discretionary foods included chocolate, candies, cookies, cake, pie, brownies, ice cream, frozen desserts, and excluded sugar-free items.

Physical activity was assessed with the PROMIS Pediatric v1.0—Physical Activity Short Form 8a [42]. This 8-item form is a valid and reliable measure of children’s lived experiences of physical activity [42]. Respondents (child self-report for ages 8 and older or a parent-proxy for younger children) reported the child’s physiologic and physical sensations (e.g., breathed hard, sweated) related to physical activity and activity frequencies. Total PROMIS T-score (range: 0 to 100) was the outcome.

The Child Media Use Questionnaire was used to assess screen time duration in minutes/day, separated by weekday and weekend use, on a typical day. This questionnaire was developed for ECHO based on the Common Sense Media surveys [43]. Screen time included educational (e.g., educational television shows, browsing informational websites) and recreational (e.g., video games) use. The questionnaire was developed before the pandemic and did not explicitly query screen time for remote schooling. Parent proxies reported screen time.

Average nightly sleep duration during the past week was assessed with the ECHO Sleep Health of Children and Adolescents Questionnaire (child self-report for ages 8 and older or a parent-proxy for younger children). Reported sleep duration less than 5 h was set to missing, and any responses more than 15 h were capped at 15 h because these were thought to be implausible values for overnight sleep duration.

### 2.4. ECHO COVID-19 Questionnaire

This parent-report questionnaire was developed in April 2020 for the ECHO Program to assess pandemic-related changes to the child’s health and behaviors. Parents’ perceived changes in children’s behaviors were assessed using 5 multiple-choice questions querying the effect of the pandemic on eating, physical activity, educational screen time, non-educational screen time, and sleep (“Compared with before the COVID-19 outbreak, how much is the child now doing the following?”) There were 3 response options: less, the same amount, or more. This questionnaire was administered concurrently with the health behavior assessments.

### 2.5. Statistical Analysis

Participant characteristics were tabulated in the full sample and by cohort. To compare unadjusted changes in health behaviors between the pre-pandemic and pandemic time periods, paired t-tests were used for continuous outcomes (physical activity, screen time, and sleep) and paired McNemar’s chi-squared test for categorical outcomes (SSBs and discretionary foods).

To examine the association between sociodemographic characteristics and behavior change, linear regression was used for continuous outcomes (physical activity T-score, averaged weekday/weekend screen time, and sleep duration) and ordinal logistic regression was used for categorical outcomes (SSBs and discretionary foods). Models were adjusted for child age, sex assigned at birth, race, ethnicity, pre-pandemic health behaviors, household annual income, and highest level of formal maternal education. We also adjusted for cohort site to account for differences between cohorts that were not accounted for in the above list of covariates, such geographic variation in the extent of pandemic-related public health precautions during the data collection period. Children were included if the child or a parent-proxy reported the child’s diet, physical activity, screen time, or sleep pre-pandemic (1 July 2019–15 March 2020) and during the pandemic (1 December 2020–30 April 2021). 

## 3. Results

This study included 347 parent-child dyads. On average, 1.2 years occurred between the baseline and pandemic measures of the health behaviors. 93% of pre-pandemic health behaviors and 100% of pandemic health behaviors were assessed during the school year. Forty-seven percent of the children were female (*n* = 163). The mean age at baseline was 7 years (Table 1). Overall, 62% of the children were non-Hispanic White, but this varied by cohort (range: 32% to 81%). Most mothers had a bachelor’s degree or higher. Participants’ area of residence was 72% metropolitan, 9% micropolitan, 2.5% small town, and 16% rural.

**Figure 1:**
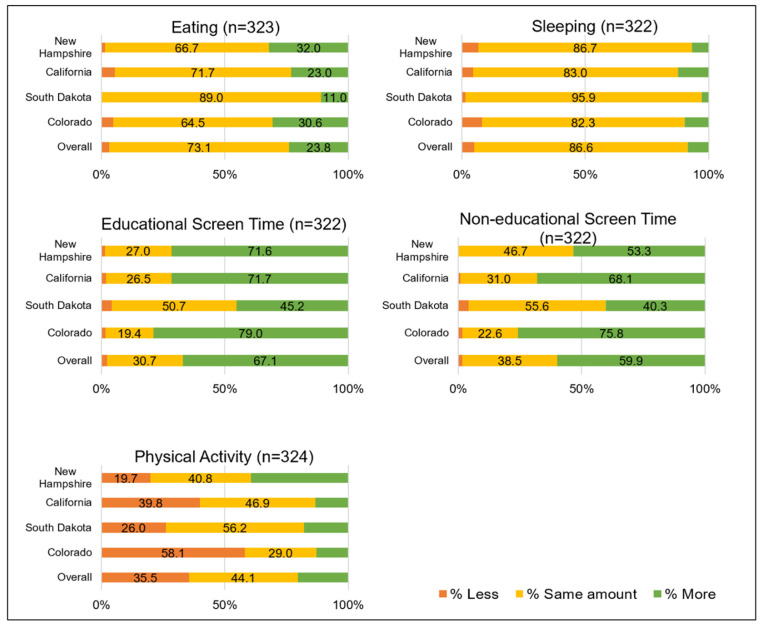
Perceived changes in children’s health behaviors during the COVID-19 pandemic among parents of children ages 4–12 years in 4 cohorts participating in the Environmental influences on Child Health Outcomes Program. Results are presented for the full sample and by recruitment site.

Figure 1 shows parents’ perceived changes in their child’s behaviors during the pandemic compared with before the pandemic. In the full sample, most parents reported no change in the amount of sleep (86.6%) and eating (73.1%). For physical activity, 40.6% of parents perceived a decline during the pandemic. For screen time, 67.1% of parents perceived an increase in educational activities and 59.9% perceived an increase in non-educational activities.

Table 2 shows the unadjusted changes in reported health behaviors between the pre-pandemic and pandemic time periods. Total screen time increased on weekdays (3.0 vs. 5.4 h, *p* ≤ 0.001) and weekends (2.5 vs. 5.3 h, *p* ≤ 0.001). No overall changes were detected in daily SSB intake (*p* = 0.265), discretionary food intake (*p* = 0.933), total activity score (*p* = 0.154), and sleep duration (*p* = 0.086).

Table 3 shows the association between sociodemographic factors and changes in SSB and discretionary food intake from pre-pandemic to pandemic. Compared with non-Hispanic White children, Hispanic children had a higher odds of changing categories of SSB intake (OR 3.31, 95% CI 1.32, 8.31). For both SSB and discretionary food intake, children who consumed these items less than once per week prior to the pandemic were the most likely to change consumption during the pandemic. Older children were more likely to change consumption of discretionary foods (OR 1.39 per year of age, 95% CI 1.07, 1.80).

Table 4 presents the association between sociodemographic factors and change in physical activity, screen time, and sleep duration. Changes in total daily screen time were largest for older children (1.67 h per year of age, 95% CI 0.25, 3.10). Median screen time increased 1.3 h per day for non-Hispanic White children, 2.3 h per day for Hispanic children, and 5.3 h per day for non-Hispanic Black children (unadjusted, data not shown). Children with longer pre-pandemic screen time duration had smaller changes (−0.63 h less change for each hour longer duration pre-pandemic, 95% CI −0.78, −0.49).

Compared with younger children, older children had a smaller average change in sleep duration (−1.04 h less change per hour longer sleep duration pre-pandemic, 95% CI: −1.47, −0.61). Results differed by pre-pandemic sleep duration; among children who met the recommended sleep recommendations pre-pandemic (at least 10 h for ages 4–5; at least 9 h for ages 6–12) [44], no change was observed in median sleep duration; however, among children who did not meet sleep recommendations pre-pandemic, a median increase of 30 min was found in sleep duration. Children with a higher physical activity score pre-pandemic had smaller changes in activity (−0.59 lower T-score per each unit higher T-score pre-pandemic, 95% CI −0.71, −0.47). 

## 4. Discussion

This study documented changes in children’s health behaviors pre-pandemic compared with during the pandemic and explored the sociodemographic correlates of change. Screen time duration increased substantially in the full sample. In addition, our findings suggest that the pandemic may have exacerbated disparities in some, but not all, health behaviors. Increases in screen time were more pronounced among children of Hispanic ethnicity and Black race. Sleep duration increased, but only among children whose sleep duration was below the recommended range pre-pandemic. Dietary changes were more pronounced among children of Hispanic ethnicity and older children.

Several findings are consistent with previous studies conducted outside the U.S. Increases in screen time are well-documented among school-aged children in multiple countries [13,14,19]. The present study showed a similar trend among U.S. children and is one of the first to report increases that persisted into 2021. This is important because screen time has been linked to child health outcomes, including adiposity and psychological well-being [45,46]. One plausible explanation for the disparities in screen time by race and ethnicity is that families who were Black and Hispanic may have experienced greater pandemic-related hardships than non-Hispanic White families [47]. in ways that influenced screen time. For example, families experiencing greater hardship may have had additional challenges coping with public health precautions that limited access to activities that do not involve screen use (e.g., safe places for physical play) [48]. Only minor changes in sleep duration were found in the study by Burkart and colleagues among school-aged children in the southeastern U.S., and no changes occurred among the overall sample reported here [19]. The 30-min increase in sleep duration among children who did not meet sleep recommendations pre-pandemic is consistent with findings among children in Asia during the lockdown period [15,18]. Much of the sociodemographic patterning observed among U.S. children was similar to that in Europe: income, education, and sex were not notable predictors of change [13,19].

Despite this alignment with other studies, several findings were unexpected. The absence of an overall change in SSBs and discretionary foods was surprising. Other studies reported increased intake of these foods and increases in the availability of processed foods in the home, snacking frequency and quality, and “pandemic baking” [23,49,50,51,52]. These inconsistencies may be due to differences in the timing of the assessments. The pandemic is a dynamic period, and it is possible that dietary behaviors observed in the initial lockdown period returned to pre-pandemic levels by 2021. If so, the present study provides novel data that these initial diet changes may be temporary. Another unexpected finding was the absence of change in physical activity measured by the PROMIS questionnaire. This was unexpected because activity decreased among European children [9,13,14]. and many parents in the present study perceived that their children’s activity declined during the pandemic. One possible explanation is that perceived changes assessed with the ECHO COVID-19 questionnaire included the intensity and duration of activity, whereas repeated assessments with the PROMIS questionnaire only queried respondents about activity frequency. Changes in the intensity and duration of activity should be investigated directly, ideally using objective tools, such as accelerometry. Additionally, physical activity was less affected among children with higher activity levels pre-pandemic; thus, studies in more sedentary samples of children could be more likely to show a decrease in activity during the pandemic.

More research is needed to identify plausible pathways by which the pandemic shaped children’s behaviors. Potential mediators are environmental factors that (1) were linked to children’s diet pre-pandemic and (2) changed during the pandemic: food insecurity, use of food pantries, processed food availability, snacking, and frequency of grocery shopping and home prepared meals [23,51,53,54]. Consistent daily routines, organized sports, and time spent outdoors were altered at some points during the pandemic and have been linked to children’s activity and screen time [54,55]. Online physical education opportunities emerged in the pandemic and were linked to increased activity [55], but their utility as an intervention tool requires further study. For sleep, shifts occurred in bedtime and wake-up time, sleep disturbance and sleep arrangement, although their effect on sleep duration during the pandemic is unclear [15,54].

This study had several strengths. The ECHO Program was uniquely positioned to measure behavior changes from the pre-pandemic to during the pandemic. The implementation of the ECHO protocol in 2019 prior to the emergence of the pandemic provided existing infrastructure for standardized and remote data collection in the pre-pandemic period and during the pandemic. The study design included recruitment sites in three U.S. Census regions (West, Midwest, and Northeast) and urban and rural areas, and thus complements a similar study that included children in the Southern U.S. [19] Further, most previous studies only measured behavior changes early in the pandemic. The present study provides insight into the effects of social and economic disruption after the initial lockdown period. Nevertheless, the pandemic is a dynamic period and data collected during a 5-month period of the pandemic in this study may not be representative of the entire pandemic period. Future research should continue to describe children’s health behaviors at other pandemic time points.

This study also had limitations. First, the observed associations between health behaviors and child race and ethnicity should be interpreted with caution. Racial and ethnic categorizations are social constructs related to many underlying characteristics of individuals and societies, such as neighborhood deprivation and systemic racism, neither of which were measured here [56]. In the present study, most children of Hispanic ethnicity or Black race resided in California and Colorado where more restrictive public health precautions (relative to New Hampshire and South Dakota) may have had an even greater impact on health behaviors. Site location was added to the statistical model to minimize potential confounding. Second, the limitations of self-reported or parent-proxy–reported health behaviors are known [57]. However, misreporting due to poor recall or social desirability is not expected to differ between the pre-pandemic and pandemic assessments; therefore, bias introduced from misreporting is expected to be minimal. Nevertheless, the screen time questionnaire was developed pre-pandemic and thus did not directly assess screen time during remote school. As a result, this study may have underestimated increases in screen time. Third, the ECHO Program is not a population-based study; accordingly, the analytic sample is not necessarily nationally representative, such as by maternal education. To enhance the generalizability of findings, this study enrolled children diverse in ethnicity, sex, and geography. Finally, the disruption of the pandemic varied over time, and the health behaviors measured in this study only reflect the period between December 2020 and April 2021. Nevertheless, the conditions during this period, including widespread community transmission of SARS-CoV-2; a combination of in-person, remote, and canceled schooling; recreational activities; and parent work may be representative of conditions outside this 5-month window.

## 5. Conclusions

Opportunities to mitigate the impact of the pandemic on child health behaviors are generally greatest for children with less healthy behaviors pre-pandemic and children who are Hispanic and non-Hispanic Black, although exceptions exist. Families may need support to prevent further declines or re-establish healthy routines for adverse behavior changes that persisted, especially screen time. Future studies should examine finer-tuned indicators of behaviors and replicate the present study design among more diverse samples of youth.

## Figures and Tables

**Table 1:** Descriptive characteristics of the analytic sample among children ages 4–12 years in 4 cohorts participating in the Environmental influences on Child Health Outcomes (ECHO) Program.

Recruitment Site	Analytic Sample	Colorado	South Dakota	California	New Hampshire
***n***	347	73	77	120	77
**Female sex,** ***n*** **(%)**	163 (47%)	37 (51%)	38 (49%)	48 (40%)	40 (52%)
**Baseline child age in years, mean (SD)**	6.9 (2.4)	8.7 (0.5)	9.5 (1.6)	4.5 (0.2)	6.3 (1.8)
**Race & ethnicity**					
Non-Hispanic White, *n* (%)	214 (62%)	39 (53%)	62 (81%)	38 (32%)	75 (97%)
Non-Hispanic Black, *n* (%)	18 (5%)	13 (18%)	<5	5 (4%)	<5
Non-Hispanic Other, *n* (%)	46 (13%)	6 (8%)	13 (17%)	25 (21%)	<5
Non-Hispanic Asian	14 (4%)	<5	<5	12 (10%)	<5
Non-Hispanic American Indian/Alaska Native	12 (3%)	<5	11	<5	<5
Non-Hispanic Multiple/Other	20 (6%)	<5	<5	12 (10%)	<5
Hispanic all races, *n* (%)	69 (20%)	15 (21%)	<5	52 (43%)	<5
**Maternal education, *n* (%)**					
High school degree, GED or equivalent or less	21 (6%)	8 (10%)	5 (7%)	<5	<10
Some college, no degree; Associate degree; trade school	98 (29%)	18 (25%)	23 (30%)	42 (35%)	15 (22%)
Bachelor’s degree	113 (33%)	18 (25%)	31 (40%)	43 (36%)	21 (31%)
Master’s degree; professional or doctorate degree	106 (31%)	29 (40%)	18 (23%)	33 (28%)	26 (38%)
Missing	9				9
**Annual household income ^1^**					
<$30,000	21 (7%)	<5	7 (10%)	7 (6%)	<5
$30,000–$49,999	27 (8%)	7 (11%)	7 (10%)	7 (6%)	<10
$50,000–$74,999	45 (15%)	9 (14%)	6 (8.7%)	15 (15%)	13 (20%)
$75,000–$99,999	58 (19%)	10 (16%)	15 (22%)	18 (16%)	15 (23%)
≥$100,000	156 (51%)	32 (52%)	34 (49%)	62 (56%)	27 (42%)
Missing	40	10	8	9	13

^1^ For the Colorado and California cohorts, household income was assessed pre-pandemic; for the North Dakota and New Hampshire cohorts, household income was only measured during the pandemic.

**Table 2:** Change in health behaviors between pre-pandemic and pandemic among children ages 4–12 years in the 4 cohorts participating in the Environmental influences on Child Health Outcomes Program (*n* = 347).

Outcomes	Pre-Pandemic ^1^	Pandemic ^2^	*p* Value	% of Children Who Increased Behavior during the Pandemic ^5^
**Dietary intake, *n* (%)**	**237**			
**Sugar-sweetened beverages** ^3^				
Less than once per week	132 (56%)	136 (57%)	0.265	19 (14%)
Once a week	25 (11%)	16 (7%)		6 (24%)
Twice a week	17 (7%)	27 (11%)		<5
3–4 times a week	20 (8%)	16 (7%)		8 (40%)
5–6 times a week	12 (5%)	16 (7%)		6 (50%)
Once per day or more	31 (13%)	26 (11%)		-
**Discretionary food** ^4^				
Less than once per week	24 (11%)	30 (13%)	0.933	12 (50%)
Once a week	17 (7%)	13 (6%)		11 (65%)
Twice a week	25 (11%)	31 (13%)		13 (52%)
3–4 times a week	41 (17%)	42 (18%)		15 (37%)
5–6 times a week	41 (17%)	40 (17%)		15 (37%)
Once per day or more	89 (38%)	81 (34%)		-
**Physical activity**	**233**			
**Total score, mean ± SD**	51.0 ± 7.9	50.4 ± 7.1	0.154	32 (14%)
**Screen time, h/day, median [IQR]**	**211**			
Total weekday duration	3.0 (1.8, 5.2)	5.4 (3.6, 8.9)	<0.001	142 (67%)
Total weekend duration	2.5 (1.5, 4.5)	5.3 (3.0, 8.7)	<0.001	120 (57%)
Total averaged duration	4.0 (2.5, 6.3)	5.6 (3.6, 8.2)	<0.001	136 (65%)
Weekday, educational	0.0 (0.0, 0.5)	1.0 (0.0, 3.0)	<0.001	105 (50%)
Weekend, educational	0.0 (0.0, 0.2)	0.0 (0.0, 0.5)	0.011	40 (19%)
Weekday recreational	2.2 (1.2, 4.3)	3.6 (2.2, 6.4)	<0.001	120 (57%)
Weekend, recreational	3.5 (2.2, 6.0)	5.3 (3.0, 8.0)	<0.001	114 (54%)
**Sleep duration, h/day, median [IQR]**	**222**			
Sleep duration	10.0 (9.0, 10.0)	9.8 (8.8, 10.0)	0.086	45 (20%)

^1^ 1 July 2019–15 March 2020; ^2^ 1 December 2020–30 April 2021; ^3^ Sugar-sweetened beverages include regular soft drinks; sweetened coffee, tea, or fruit drinks; and sports or energy drinks. Excludes flavored milks, 100% fruit juices, and artificially sweetened beverages; ^4^ Discretionary foods include chocolate, candies, cookies, cake, pie, brownies, ice cream, other frozen desserts. Excludes sugar-free discretionary food; ^5^ *n* (%) of children for whom increases in each health behavior were observed in the pandemic period compared with the pre-pandemic period. Increase in behavior is defined as follows: increasing ≥1 category (diet); increasing ≥1 SD (physical activity); increasing ≥1 h (screen time and sleep duration).

**Table 3:** Associations between sociodemographic characteristics and changes in dietary intake among children ages 4–12 years in 4 cohorts participating in the Environmental influences on Child Health Outcomes Program.

	Sugar-Sweetened Beverages OR (95% CI) ^1^	Discretionary Food OR (95% CI) ^1^
	***n*** **= 206**	***n*** **= 206**
**Age**	1.28 (0.90, 1.81)	1.39 (1.07, 1.80) *
**Sex, male**	1.08 (0.59, 1.96)	0.71 (0.42, 1.21)
**Race/Ethnicity**		
Non-Hispanic White	**REF**	**REF**
Non-Hispanic Black	1.37 (0.27, 6.92)	1.12 (0.24, 5.21)
Non-Hispanic Other	1.33 (0.48, 3.72)	1.45 (0.58, 3.62)
Hispanic	3.31 (1.32, 8.31) *	0.87 (0.38, 1.97)
**Income**		
<$50,000	**REF**	**REF**
$50,000–$74,999	0.49 (0.16, 1.45)	2.36 (0.88, 6.33)
$75,000–$99,999	1.18 (0.39, 3.58)	1.71 (0.64, 4.59)
$100,000 or more	0.75 (0.26, 2.15)	2.11 (0.84, 5.30)
**Education**		
High school degree, GED or equivalent or less	**REF**	**REF**
Some college/associate degree/trade school	1.25 (0.28, 5.57)	2.10 (0.59, 7.52)
Bachelor’s degree	1.08 (0.23, 5.02)	2.61 (0.70, 9.69)
Master’s degree; professional or doctorate degree	0.65 (0.13, 3.17)	2.20 (0.58, 8.32)
**Pre-pandemic outcome**		
Less than once per week	**REF**	**REF**
Once a week	0.12 (0.04, 0.36) **	0.58 (0.17, 2.01)
Twice a week	0.08 (0.02, 0.29) **	0.35 (0.11, 1.15)
3–4 times a week	0.21 (0.05, 0.81) *	0.10 (0.03, 0.30) **
5–6 times a week	0.83 (0.18, 3.93)	0.07 (0.02, 0.22) **
Once per day or more	0.02 (0.00, 0.07) **	0.04 (0.01, 0.12) **

^1^ Odds ratios (95% CI) from ordinal regression models represent the odds of changing outcome categories. Models are adjusted for covariates shown in table and cohort. GED, general educational development. * *p* < 0.05; ** *p* < 0.01.

**Table 4:** Associations between sociodemographic characteristics and changes in physical activity, screen time, and sleep duration among children ages 4–12 in 4 cohorts participating in the Environmental influences on Child Health Outcomes Program.

	Change in Child Health Behaviors ^1^		
	Physical Activity PROMIS T-Score	Screen Time	Sleep Duration
	***n* = 208**	***n* = 203**	***n* = 196**
**Age**	−1.23 (−3.43, 0.98)	1.67 (0.25, 3.10) *	−1.04 (−1.47, −0.61) **
**Sex, male**	0.65 (−1.28, 2.57)	−0.93 (−1.97, 0.11)	0.02 (−0.30, 0.34)
**Race/Ethnicity**			
Non-Hispanic White	**REF**	**REF**	**REF**
Non-Hispanic Black	−3.30 (−7.74, 1.14)	4.02 (1.56, 6.47) **	0.06 (−0.76, 0.88)
Non-Hispanic Other	−0.33 (−3.39, 2.74)	2.09 (0.56, 3.63) **	0.09 (−0.38, 0.56)
Hispanic	0.46 (−2.31, 3.22)	1.56 (0.06, 3.07) *	0.41 (−0.05, 0.87)
**Income**			
<$50,000	**REF**	**REF**	**REF**
$50,000–$74,999	1.80 (−1.70, 5.30)	−0.43 (−2.31, 1.46)	−0.13 (−0.73, 0.46)
$75,000–$99,999	4.99 (1.37, 8.61) **	0.32 (−1.65, 2.29)	0.30 (−0.29, 0.90)
$100,000 or more	2.26 (−1.31, 5.82)	−0.88 (−2.72, 0.95)	0.45 (−0.11, 1.01)
**Education**			
High school degree, GED or equivalent or less	**REF**	**REF**	**REF**
Some college/associate degree/trade school	3.20 (−2.03, 8.42)	−1.46 (−4.68, 1.77)	−0.02 (−1.07, 1.03)
Bachelor’s degree	4.91 (−0.35, 10.16)	−1.78 (−5.04, 1.49)	0.16 (−0.90, 1.22)
Master’s degree; professional or doctorate degree	3.46 (−1.98, 8.91)	−2.58 (−5.92, 0.75)	0.14 (−0.95, 1.23)
**Pre-pandemic behavior** ^**2**^	−0.59 (−0.71, −0.47) **	−0.63 (−0.78, −0.49) **	−0.56 (−0.70, −0.42) **

^1^ Beta coefficients (95% CI) from these regression models represent the change in outcome in hours (screen time and sleep) or total score (physical activity). Models are adjusted for covariates shown in the table and cohort. ^2^ Pre-pandemic screen time was included in the screen time model; pre-pandemic sleep was included in the sleep model; and pre-pandemic physical activity was included in the physical activity model. GED, general educational development. * *p* < 0.05; ** *p* < 0.01.

## Data Availability

The datasets for this manuscript are not publicly available because, per the NIH-approved ECHO Data Sharing Policy, ECHO-wide data have not yet been made available to the public for review/analysis. Requests to access the datasets should be directed to the ECHO Data Analysis Center, ECHO-DAC@rti.org.
